# A Machine Learning Algorithm to Predict Medical Device Recall by the Food and Drug Administration

**DOI:** 10.5811/westjem.21238

**Published:** 2024-11-21

**Authors:** Victor Barbosa Slivinskis, Isabela Agi Maluli, Joshua Seth Broder

**Affiliations:** * Duke University, Pratt School of Engineering, Department of Biomedical Engineering, Durham, North Carolina; † Duke University, Trinity College of Arts and Sciences, Department of Chemistry, Durham, North Carolina; ‡ Duke University School of Medicine, Department of Emergency Medicine, Durham, North Carolina

## Abstract

**Introduction:**

Medical device recalls are important to the practice of emergency medicine, as unsafe devices include many ubiquitous items in emergency care, such as vascular access devices, ventilators, infusion pumps, video laryngoscopes, pulse oximetry sensors, and implantable cardioverter defibrillators. Identification of dangerous medical devices as early as possible is necessary to minimize patient harms while avoiding false positives to prevent removal of safe devices from use. While the United States Food and Drug Administration (FDA) employs an adverse event reporting program (MedWatch) and database (MAUDE), other data sources and methods might have utility to identify potentially dangerous medical devices. Our objective was to evaluate the sensitivity, specificity, and accuracy of a machine learning (ML) algorithm using publicly available data to predict medical device recalls by the FDA.

**Methods:**

We identified recalled medical devices (RMD) and non-recalled medical devices (NRMD) using the FDA’s website and online database. We constructed an ML algorithm (random forest regressor) that automatically searched Google Trends and PubMed for the RMDs and NRMDs. The algorithm was trained using 400 randomly selected devices and then tested using 100 unique random devices. The algorithm output a continuous value (0–1) for recall probability for each device, which were rounded for dichotomous analysis. We determined sensitivity, specificity, and accuracy for each of three time periods prior to recall (T-3, 6, or 12 months), using FDA recall status as the reference standard. The study adhered to relevant items of the Standards for Reporting Diagnostic accuracy studies (STARD) guidelines.

**Results:**

Using a rounding threshold of 0.5, sensitivities for T-3, T-6, and T-12 were 89% (95% confidence interval [CI] 69–97), 90% (95% CI 70–97), and 75% (95% CI 53–89). Specificity was 100% (95% CI 95–100) for all three time periods. Accuracy was 98% (95% CI 93–99) for T-3 and T-6, and 95% (95% CI 89–99) for T-12. Using tailored thresholds yielded similar results.

**Conclusion:**

An ML algorithm accurately predicted medical device recall status by the FDA with lead times as great as 12 months. Future research could incorporate longer lead times and data sources including FDA reports and prospectively test the ability of ML algorithms to predict FDA recall.

Population Health Research Capsule What do we already know about this issue?

*Identification of dangerous medical devices by the US Food and Drug Administration is essential to minimize patient harms while avoiding unnecessary recalls.*

What was the research question?

*We evaluated the performance of a machine learning algorithm to predict recalls using publicly available data.*

What was the major finding of the study?

*Sensitivity for recall was 75% (95% CI 53–89) with specificity 100% (95% CI 95–100) with a 12-month lead time.*

How does this improve population health?

*Machine learning algorithms might risk-stratify devices for further FDA investigation, improving resource allocation while allowing safe devices to remain in use.*

## INTRODUCTION

In our institution we have encountered multiple safety events associated with medical devices including chest tubes and vascular catheters, which we reported to the manufacturers and the US Food and Drug Administration (FDA).^1^ ^–^ ^3^ Our experience inspired an interest in understanding the FDA recall process and developing methods to improve the efficiency of post-market, device safety evaluation. Medical device recalls are important to the practice of emergency medicine, as they affect patients, emergency physicians and other practitioners, health systems, and manufacturers. Unsafe devices and recalls include many ubiquitous items in emergency care, such as vascular access devices, ventilators, infusion pumps, video laryngoscopes, pulse oximetry sensors, and implantable cardioverter defibrillators.^4^

Appropriate recall of dangerous devices at the earliest possible time limits further patient harm, while failing to recall an unsafe device exposes patients to potential injury or death. Inappropriate or excessively conservative device recalls harm patients by depriving them of device benefits; even appropriate recalls may leave no clinical alternative, creating potential harm. Product recalls impose a considerable burden on health systems and manufacturers by incurring costs of investigations for potential recalls, recalling those devices, and developing and buying substitute devices to fill a post-recall vacuum. Regulatory agencies face the challenges of investigative costs with finite resources, ongoing harms prior to recall (or even post-recall, as some products such as implanted devices may still be in use), and costs related to removing those devices and evaluating and approving alternatives. Therefore, developing and investigating automated methods to predict such recalls are a crucial area of study.

An optimal monitoring and recall system would identify all dangerous devices without errors (ie, no false positives or negatives) as early as possible, with human intervention required only to validate the findings. In the United States, the FDA approves medical devices and conducts recalls of unsafe devices.^4^ Currently, the FDA has a passive, post-market approval system (MedWatch) where patients, clinicians (including emergency physicians), and healthcare systems can submit reports of medical device-associated adverse events, and a publicly available, searchable monitoring database MAUDE (Manufacturer and User Facility Device Experience), where reports are logged.^5^ ^,^ ^6^ The FDA acknowledges that the accuracy of submitted data and causal relationships are unknown.^7^ Most device recalls occur voluntarily by manufacturers and are governed by Title 21 of the Code of Federal Regulations (CFR) 7.^8^

The FDA also evaluates medical device recalls through 21 CFR 810 and 21 CFR 806.^9^ Part 810 outlines the recall process, including evaluating health risks and defining the recall’s extent. This section designates devices into three classes based on potential risk severity. Class I includes devices that have the potential to cause serious risks of harm or death; II designates those that may cause temporary or reversible risks and pose a slight chance of more serious harm or death; and III includes devices not likely to cause health problems or injury. Part 806 focuses on reporting requirements for manufacturers initiating a recall or correction. Manufacturers must report any device correction or removal to reduce health risks, including the reason for the recall and the total quantity produced. Together, 21 CFR 7, 810, and 806 provide a framework for identifying and rectifying issues, with the goal of ensuring medical devices’ ongoing safety and effectiveness in the marketplace. The FDA considerations include the nature and potential health risk of the device, the extent and cause of the defect, the likelihood of occurrence, the manufacturer’s recall strategy, the number of affected products, the distribution pattern, and the level of hazard presented to patients.

From 2018–2023, the FDA approved or cleared more than 250 medical devices.^10^ The FDA receives approximately one million reports annually through the MedWatch and MAUDE systems.^7^ From September 2018–September 2023, 234 serious device recalls were issued by the FDA, with thousands of additional recalls during that period.^4^ ^,^ ^11^ Among devices reaching the market between 2008–2017, 10.7% of devices with 510(k) clearance (FDA pre-market review process) and 27.1% of those with pre-market approval were recalled.^12^ To address recalls and its other missions, the FDA has approximately 18,000 employees, including 1,887 employed by the Center for Devices and Radiological Health in 2020.^13^ ^,^ ^14^ Safety monitoring is thus a logistical challenge because of the disparity between resources and devices to be monitored, as well as the labor-intensive job of evaluating each device’s merit for recall.

An automated tool to assist in device risk stratification would be invaluable, and machine learning (ML) could be employed for this purpose. Machine learning is a field of artificial intelligence in which large datasets are used to train an algorithm to categorize or analyze new data. ML follows two sequential phases. In the initial training phase, labeled data is supplied to enable the algorithm to learn to differentiate between relevant classes (eg, “this is a dog, this is a cat,” or “this is a dangerous medical device, this is not”). In a subsequent testing phase, the algorithm is presented with new, unlabeled data and asked to differentiate between classes (eg, “is this a cat or is it a dog?”; “will this medical device be recalled or not?”).

Large data sources external to the FDA system might provide useful signals of device dangers for use in ML algorithms. PubMed is a free database with more than 37 million citations of biomedical and life science literature, developed and maintained by the National Center for Biotechnology Information; reports of adverse events involving medical devices might be reported here.^15^ Google Trends is a free tool that quantifies the frequency of search terms on Google Search, YouTube, Google News, Google Shopping, and Google Images over time.^16^ The FDA itself provides a list of approved and recalled medical devices.^4^

We developed and evaluated an ML algorithm to predict medical device recalls by the FDA using publicly available data. We discuss its potential value for patient safety as well as challenges and limitations of using ML for risk stratification of medical devices.

## METHODS

We conducted a retrospective, diagnostic case-control study. Actual FDA-recall status of devices was determined from FDA sources as described below. An ML algorithm was then trained to predict the probability of FDA recall using data from PubMed and Google Trends. In the testing phase, the algorithm was blinded to the FDA recall status of devices and produced a prediction of probability of recall, which was then compared to actual recall status. We tested the ability of the algorithm to predict recall with lead times of three, six, and 12 months before an actual recall by limiting the algorithm’s access to search data for the corresponding time period. Our methods were consistent with relevant elements of the Standards for Reporting Diagnostic Accuracy Studies (STARD) guidelines, such as definitions of the index test and reference standard, estimates of diagnostic accuracy and precision, analyses of variability in diagnostic accuracy, blinding, and potential sources of bias (Appendix 1).^17^

### Device Definitions and Identification

Two categories were defined: recalled medical devices (RMD) (“cases”) and non-recalled medical devices (NRMD) (“controls”). We identified RMD through the FDA’s webpage listing serious recalls.^4^ The NRMD were identified by being in the medical market without being recalled by checking against the FDA list for RMD. Devices were selected from January 1, 2019–September 17, 2023, excluding repeated devices or severe acute respiratory syndrome-related coronavirus-2 (SARS-CoV-2) tests. We excluded SARS-CoV-2 tests as they were in the market for very few years and could produce anomalous Google Trends and PubMed data due to their association with COVID-19. All devices included in the study were randomly selected from the pools of identified RMD and NRMD.

### Time Frame/Lead Time for Prediction

Recognizing that the FDA investigations and other processes culminating in a device recall may require a period of months or years to complete following the first reports of potential harm, we sought to develop a forecasting tool that could predict recalls up to 12 months before their occurrence. We explored data in three different sets, each lasting five years and ending months prior to the actual FDA recall: three months before recall (T-3); six months before recall (T-6); and 12 months before recall (T-12). Each of the three sets thus included five years of data.

### Device Data

For each RMD and NRMD identified in the FDA webpage, PubMed and Google Trends were automatically searched by the ML algorithm using the device names (Appendix 2) and the three date ranges described above (T-3, T-6, T-12) relative to the date of recall. We included queries without Google Trends or PubMed data in the training and testing dataset to avoid selection bias.

### Machine Learning Algorithm

We built an ML algorithm on Python 3.8.5 (Python Software Foundation, Wilmington, DE) using random forest regressor, an open-source algorithm.^18^ ^–^ ^20^ This type of algorithm is used for large-volume, multivariable data and is suitable for sets with missing values, noisy data, and outliers. Random forest regressor typically uses 80% of data for training and 20% for testing.^21^ A regressor algorithm outputs a continuous decimal value between 0 and 1; in our application, 0 represents 0% likelihood of recall, and 1 represents 100% likelihood of recall. We chose a regressor over an alternative algorithm type, a classifier. A classifier sorts the output of an algorithm into categories such as “recalled” and “not recalled.” The continuous value outputs of a regressor can be converted into discrete classifications by application of threshold values, allowing additional analysis. In contrast, if a classifier had been applied, discrete data could not be converted into continuous values.

### Training and Testing Phases

**Figure 1.**
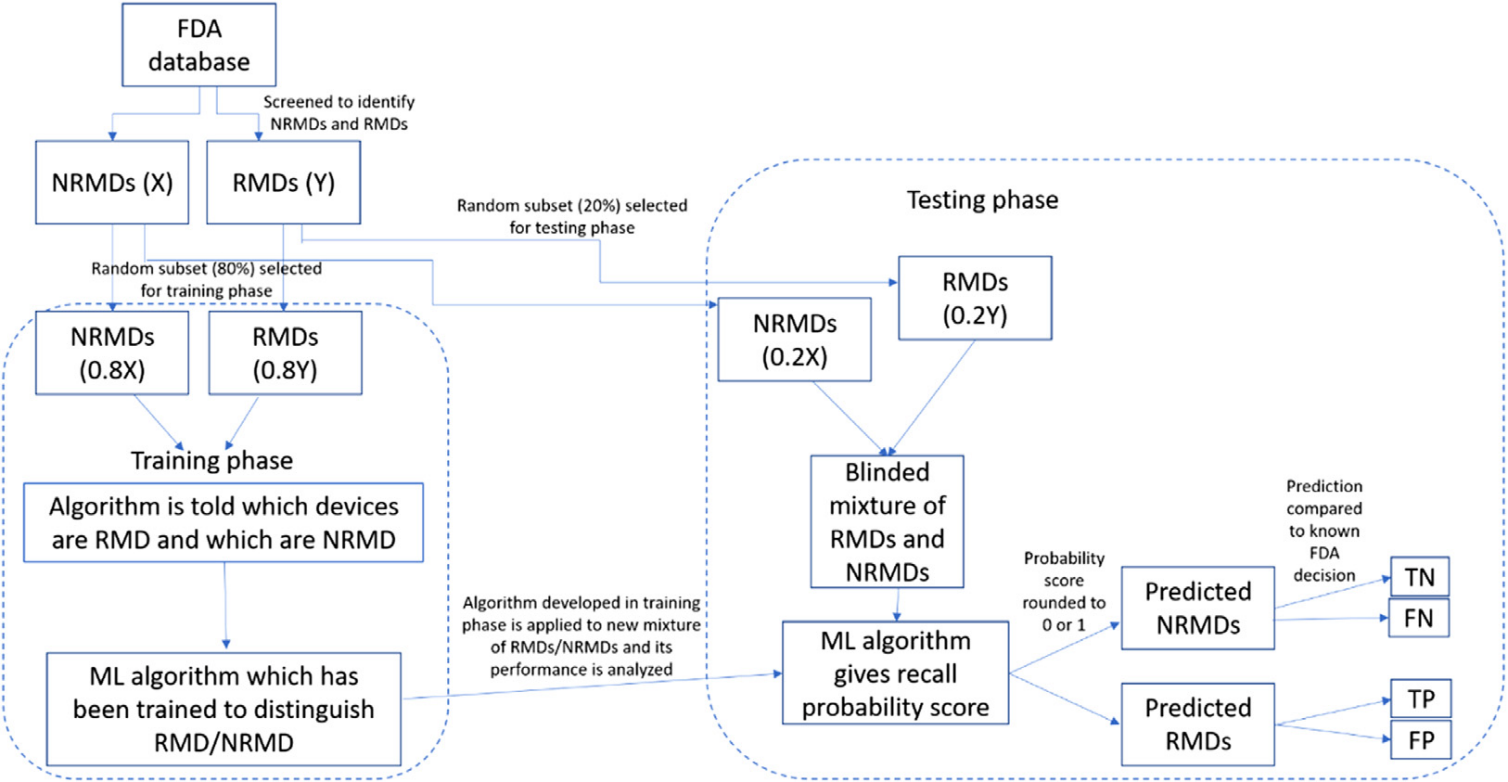
Flowchart for training and testing phases of the machine learning algorithm. Training and testing data were two unique sets with no overlap. *FDA*, US Food and Drug Administration; *FN*, false negative; *FP*, false positive; *ML*, machine learning; *NRMD*, non-recalled medical devices; *RMD*, recalled medical devices; *TN*, true negative; *TP*, true positive.

For this study, we performed a training phase, where the algorithm learned to differentiate between RMD and NRMD with 400 randomly selected training data (T-3: 81 RMD, 319 NRMD; T-6 and T-12: 80 RMD, 320 NRMD), with devices labeled for the algorithm as RMD or NRMD. In the testing phase, 100 randomly selected testing data (T-3: 19 RMD, 81 NRMD; T-6 and T-12: 20 RMD, 80 NRMD) were provided to the trained ML algorithm to assess its performance in differentiating the unlabeled devices. Training and testing data were two unique sets with no overlap. Figure 1 outlines the training and testing phases.

We converted continuous decimal prediction outputs of the ML algorithm from the testing phase to dichotomous values of zero or one using two previously published strategies^22^:

1. Pre-specified: Rounding using a threshold of 0.5, where all values < 0.5 were rounded to zero and all values ≥ 0.5 were rounded to one. This approach groups valid but potentially indeterminate values (close to 0.5) with positive (1) and negative (0) results.^22^
2. Exploratory: Rounding using thresholds determined from the ML algorithm output of the training data, described below. This approach addresses values very close to 0.5 that might have little predictive value and might be better treated as uninterpretable or inconclusive. To calculate these thresholds, training data was processed by the trained ML algorithm to yield continuous decimal predictions, which were then analyzed by their actual FDA recall status (RMD or NRMD). Using the means and one standard deviation in this fashion would be anticipated to encompass 84% of the data of a normally distributed dataset, excluding values close to 0.5 (for NRMD, 50% from data below the mean and 34% from data one SD above the mean; for RMD, 50% from data above the mean and 34% from one SD below the mean).
  a. The range of NRMD_testing_ was defined as zero to one SD above the mean output values of NRMD_training_ (0 to [mean_NRMDtraining_ + SD_NRMDtraining_]). Thus, in the testing phase, output values ≤ (mean_NRMDtraining_ + SD_NRMDtraining_) were rounded to zero.
  b. The range of RMD_testing_ was defined as one SD below the mean of the predictions for RMD_training_ to one ([mean_RMDtraining_ − SD_RMDtraining_] to 1). Thus, in the testing phase, output values ≥ (mean_RMDtraining_ − SD_RMDtraining_) were rounded to one.
  c. Intermediate values between (mean_NRMDtraining_ + SD_NRMDtraining_) and (mean_RMDtraining_ − SD_RMDtraining_) were assigned to a third category, “indeterminate.” These were not rounded and were not included in calculations requiring dichotomous outcomes.^22^

### Outcomes and Statistical Analysis

We recorded ML-algorithm-generated probability of recall and actual recall status as pre-specified outcomes. We calculated sensitivity, specificity, and accuracy as pre-specified outcomes, using pre-specified and exploratory threshold values as described above. A true positive was considered a device predicted by the ML algorithm to be recalled, which was actually recalled by the FDA. A true negative was considered a device predicted by the ML algorithm not to be recalled, which the FDA did not actually recall. A false positive was considered a device predicted by the ML algorithm to be recalled, which the FDA did not actually recall. A false negative was considered a device predicted by the ML algorithm not to be recalled, which the FDA actually recalled. We defined accuracy as (true positives + true negatives) divided by total devices. Test yield was defined as the fraction of test results included in calculation of binary outcomes (sensitivity, specificity, accuracy) after exclusion of indeterminate results.^22^

### Sample Size

Because we were investigating a novel application and data sources for an ML algorithm, we had no prior data for power or sample-size calculations. Given limited FDA resources, high specificity was prioritized to avoid wasteful investigation of devices that are, in fact, safe. For a target specificity point estimate of 100%, 80 NRMD in the testing phase would yield a 95% confidence interval (CI) 0.95–1.0.^23^ ^,^ ^24^ The algorithm’s focus was to flag potential devices that should be recalled in a sea of medical devices, most of which do not need to be recalled. Therefore, more NRMD than RMD were required, and we selected a 4:1 ratio. As described above, the ML algorithm partitioned 20% of the sample to the testing phase and 80% to the training phase. This yielded a total sample size of 400 NRMD and 100 RMD.

## RESULTS

**Figure 2.**
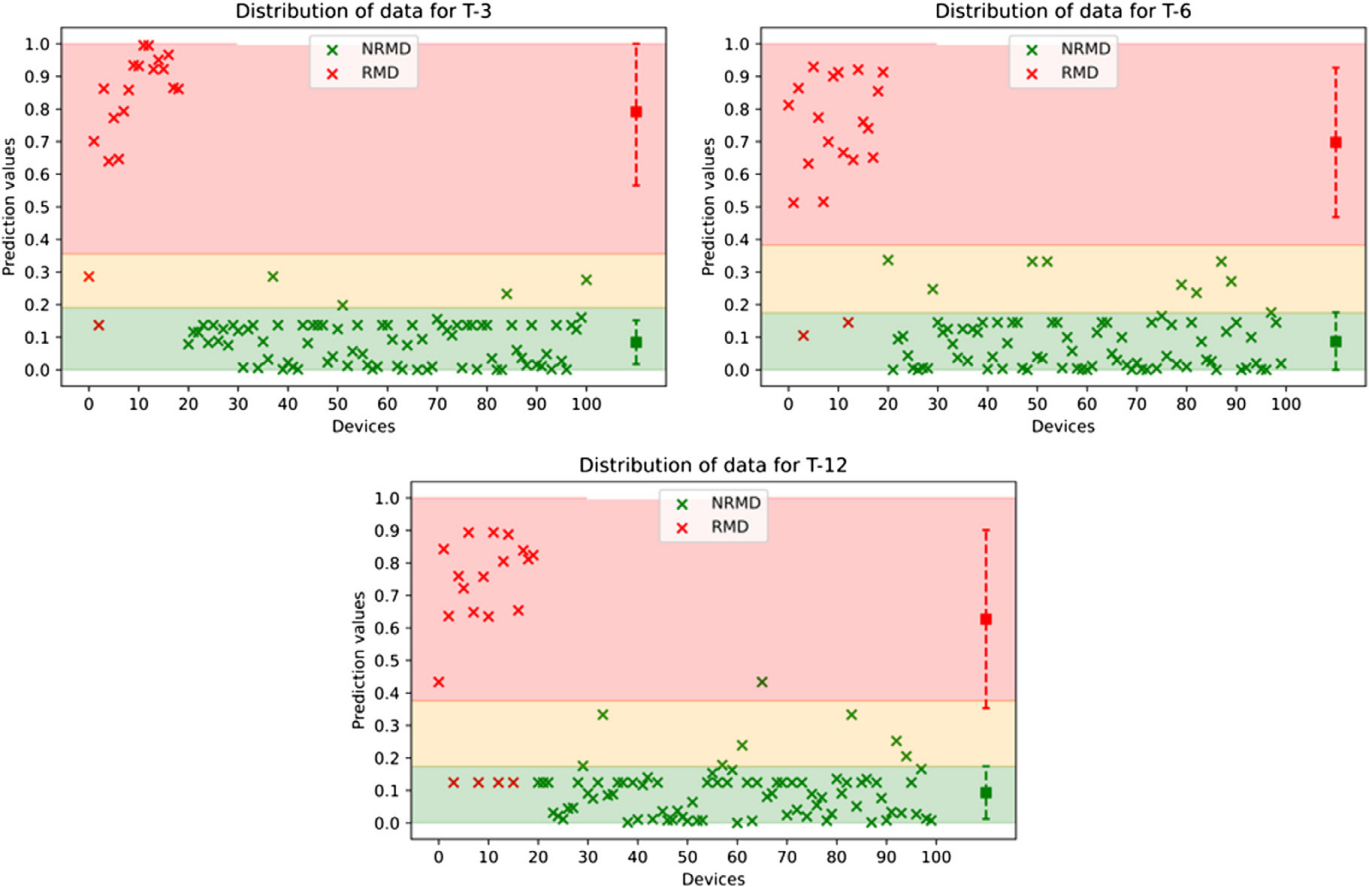
The distribution of devices in the testing dataset that were either recalled or not recalled by the US Food and Drug Administration with lead times of 3, 6, and 12 months. Red x’s represent recalled medical devices (RMD) and green x’s represent non-recalled medical devices (NRMD). The colored regions represent ranges determined from training data predictions. Thus, green x’s in the green zone are true negatives; red x’s in the red zone are true positives; green x’s in the red zone are false positives; and red x’s in the green zone are false negatives. The square represents the mean of each category; the error bars span +/− one standard deviation for the testing results.

**Figure 3.**
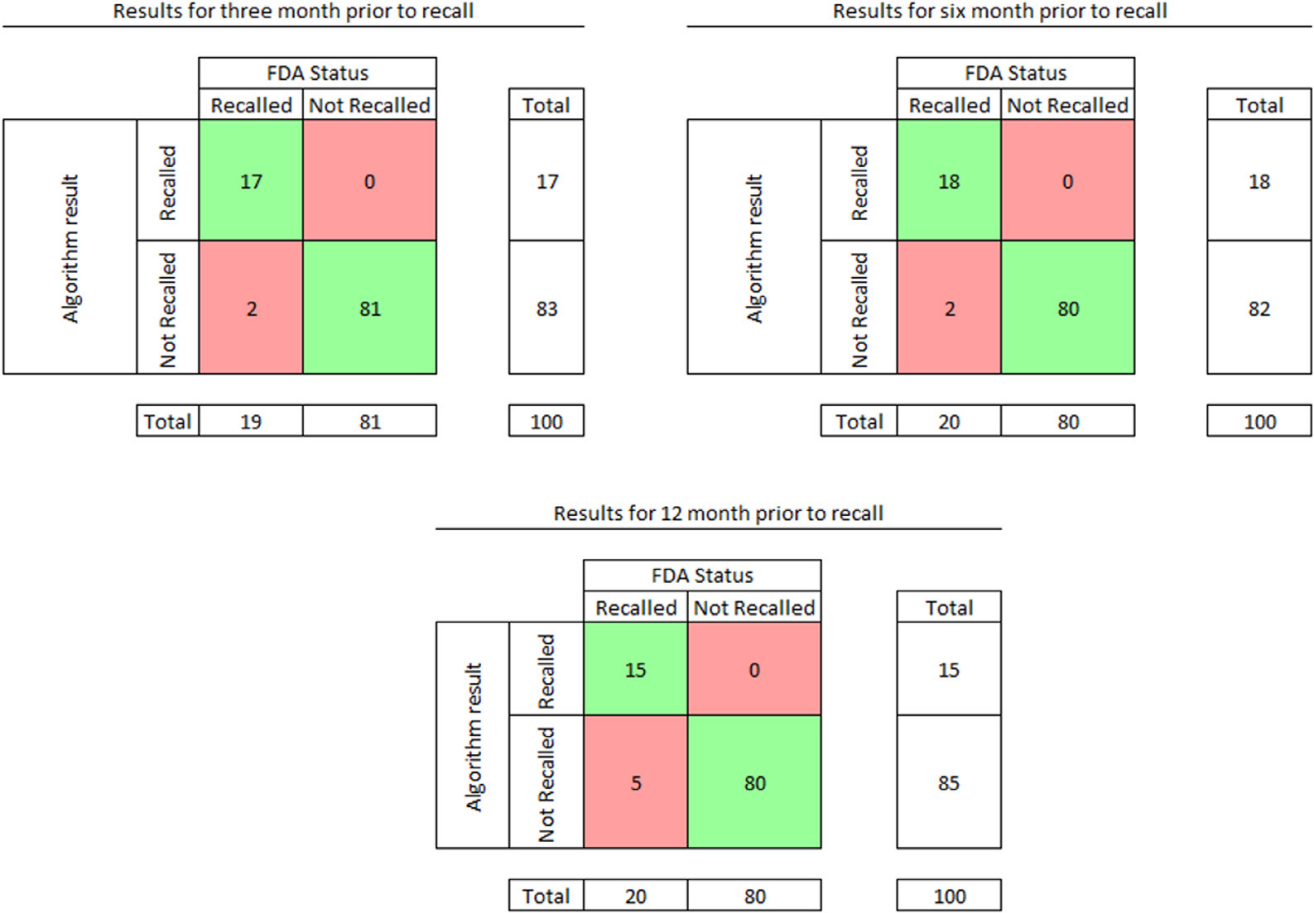
Results of the testing phase when rounding using 0.5, where green indicates true positive and true negative, red indicates false positives and false negatives. Zero false-positive results were encountered during the tested time periods ending 3, 6, or 12 months before US Food and Drug Administration (FDA) recall. False negative results were uncommon in all three time periods. All devices and recall predictions are included in the analysis.

**Figure 4.**
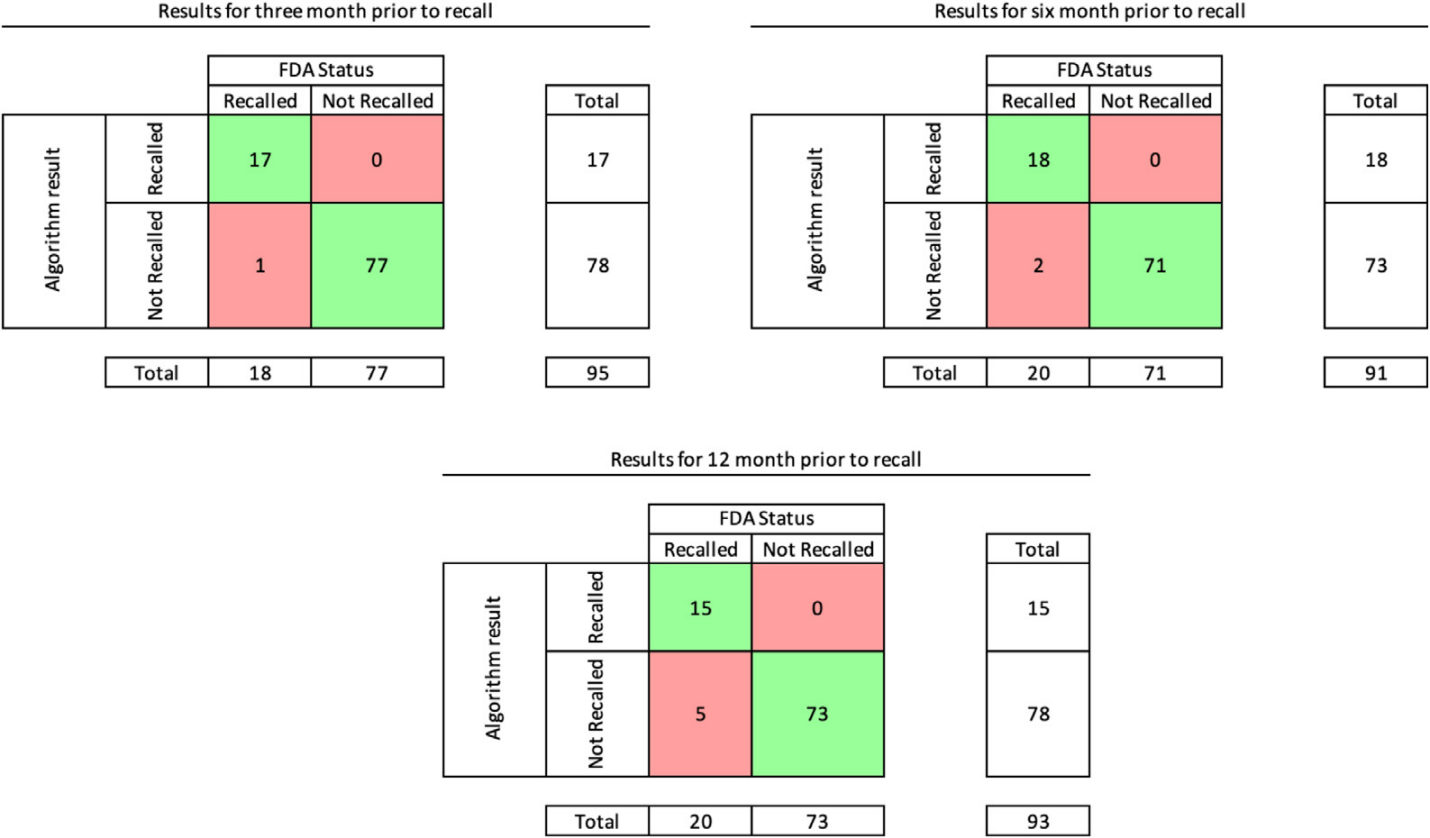
Results of the testing phase when removing the data in the indeterminate zone and then rounding all data in the green zone to 0 and all data in the red zone to 1. Green indicates true positive and true negative; red indicates false positives and false negatives. Zero false-positive results were encountered during the tested time periods (T) ending 3, 6, or 12 months before FDA recall. False negative results were uncommon in all three time periods. Test yield (the fraction of all test results used in calculating binary outcomes after removal of indeterminate values)^22^ was 95% at T-3, 91% at T-6, and 93% at T-12 months.

The ML algorithm continuous prediction values for each device from testing data are plotted in Figure 2, superimposed on threshold ranges determined from training data. The performance of the algorithm using a rounding threshold for recall probability of 0.5 is shown in Figure 3 and Table 1. The performance of the algorithm using rounding thresholds for recall probability determined from training data is shown in Figure 4 and Table 2. We excluded devices in the indeterminate (yellow) zone (Figure 2) from this analysis, with test yield reported.^22^

**Table 1.** Performance of the machine learning algorithm with lead times of 3, 6, and 12 months prior to actual recall when rounding recall probability of 0.5 and above to 1, otherwise to 0.

Lead time	Sensitivity	Specificity	Accuracy
T-3	89 (95% CI 69–97)	100 (95% CI 95–100)	98 (95% CI 93–99)
T-6	90 (95% CI 70–97)	100 (95% CI 95–100)	98 (95% CI 93–99)
T-12	75 (95% CI 53–89)	100 (95% CI 95–100)	95 (95% CI 89–98)

*T-3*, 3 months before recall; *T-6*, 6 months before recall; *T-12*, 12 months before recall; *CI*, confidence interval.

**Table 2.** Performance of the machine learning algorithm with lead times of 3, 6, and 12 months prior to actual recall when removing indeterminate devices and rounding all values that were in green to 0 and all values that were in red to 1.

Lead time	Sensitivity	Specificity	Accuracy
T-3	94 (95% CI 74–99)	100 (95% CI 95–100)	99 (95% CI 92–100)
T-6	90 (95% CI 70–97)	100 (95% CI 95–100)	97 (95% CI 91–99)
T-12	75 (95% CI 53–89)	100 (95% CI 95–100)	94 (95% CI 86–98)

*T-3*, 3 months before recall; *T-6*, 6 months before recall; *T-12*, 12 months before recall; *CI*, confidence interval.

## DISCUSSION

Our development of an ML risk-stratification tool was motivated by our experience with repeated safety events with pigtail catheters and large, vascular access devices.^1^ ^–^ ^3^ We reported these to manufacturers and the FDA. To our knowledge, the FDA responses were limited to written acknowledgments of our reports, with some of our suggested modifications incorporated by manufacturers as described below. Given the volume of reports annually to FDA, we conceived that better, automated methods for risk stratification might be needed.

Our study demonstrates the potential of an ML algorithm using publicly available, large datasets to predict medical device recalls with high sensitivity, specificity, and accuracy with lead times of three, six, and 12 months (Tables 1 and 2). The high specificity (ie, lack of false positives) with narrow confidence intervals as early as 12 months before recall indicates that devices flagged for recall are likely to be true-positive unsafe devices, worthy of further FDA investigation. The algorithm is unlikely to flag non-recalled devices for recall (false positive), an important feature when screening a large device pool consisting primarily of safe devices. A low false-positive rate may avoid unnecessary costs and resource utilization.

The high sensitivity with lead times of three and six months suggests that dangerous devices are likely to be identified by the algorithm; so devices that are rated as “not recalled” are likely safe and may not generally deserve additional FDA scrutiny without specific suspicion or concern (eg, reports of severe adverse events in MAUDE). The sensitivity of 75% with a 12-month lead time is less than we had hoped for but may be acceptable because of the high specificity (100%). Even though only three-quarters of eventually-recalled devices were flagged by the algorithm at this early time-point, all flagged devices were true positives and therefore we believe would be appropriate for further FDA investigation.

The probability of recall outputs of our ML algorithm were continuous values (Figure 2). We collapsed these into binary categories for calculation of sensitivity and specificity, a common practice for diagnostic tests. Continuous variables often result in valid but indeterminate results, which can be addressed by various strategies, each with benefits and costs—and with no single agreed-upon solution.^22^ ^,^ ^25^ For clinical diagnostic tests, different “rule in” and “rule out” thresholds are often used, with an indeterminate zone of test results recognized. Examples include brain-type natriuretic peptide, procalcitonin, and estimated glomerular filtration rate.

We applied two commonly used approaches as a means of sensitivity analysis: combining indeterminate results into positive and negative categories (Figure 3, Table 1), and removing indeterminate results (Figure 4, Table 2). Exclusion of indeterminate results can overestimate test performance, while combining inconclusive results can underestimate accuracy and may not be sensible in the intended application.^22^ For example, characterizing a device with a 51% predicted probability of recall as an RMD would potentially result in wasteful expenditure of FDA resources for investigation. Representing a device with a 49% recall probability as an NRMD might imply greater safety than warranted. In our present study, we found no statistically significant difference in test performance by excluding indeterminate results (95% CI for all measures overlap for the two analyses), and we present both analyses for transparency. The test yield^22^ was greater than 90% for all time periods, indicating that a minority of results were excluded as indeterminate values.

Device recalls can have substantial impacts in the practice of emergency medicine (and other specialties), even when appropriate. Further illustrating these diverse impacts and validating the need for enhanced tools for risk stratification is that after the initiation of our work on ML algorithms we encountered another hazardous device in our institution. An arterial catheter used routinely in our emergency departments, operating rooms, and intensive care units was noted to create a risk of arterial embolization of catheter fragments. Removing the device from circulation, replacing it with a viable alternative, and retraining hundreds of healthcare workers occurred over a period of approximately six months—before a manufacturer urgent recall was issued on May 19, 2023, and an FDA Class I recall (the most serious type) followed.^26^ ^,^ ^27^ By the date of the recall, the device had been in distribution for 4.5 years (October 26, 2018–May 10, 2023) during which time the manufacturer had received 83 complaints of related device malfunctions and 18 injuries. A total of 262,016 devices were recalled in the US. Other FDA medical device recalls relevant to emergency medicine practice in 2023 included angiography catheters (for failure to undergo sterilization), infusion pumps (for failure to detect air in line), video laryngoscopes (for stolen defective products), pulse oximetry sensors (for inaccurate readings and interference with defibrillators), implantable cardioverter defibrillators (for low or no energy output), and ventilators (for short circuits and stopping without notice).^4^

Additional literature has recently explored the impact of medical device recalls on patients and healthcare systems. In 2021, Philips Respironics recalled airway pressure devices for carcinogenic chemical emissions and significant adverse effects including respiratory distress, inflammation, hypoxia, and hypercarbia.^28^ Approximately 16 million domestic and international patients were affected by the recall, resulting in organizations, including the Mayo Clinic, developing novel protocols to ensure centralized awareness of device recalls, aid staff in visualizing their proactive approaches to the situation, and efficiently communicate when informing patients about the recall.

Medical device recalls involve costs and efforts for multiple stakeholders (including patients, physicians, health systems, manufacturers, insurers, and regulators), from removing recalled devices to developing alternatives to integrate them into the market effectively. The estimated mean development cost for a novel complex medical device is $60 million (95% CI, $27 million–$209 million) after accounting for post-approval studies. Accounting for cost of capital and failed devices, the estimated mean cost per approved device is nearly 10-fold higher: $526 million (95% CI, $207 million–$3396 million). From nonclinical trials to FDA approval, the estimated development time of novel devices is 157 months (13 years).^29^ Assessing the economic impact of medical device recalls in the broader healthcare ecosystem poses many challenges due to factors including regulatory conditions, the role of device integration into medical procedures, and the temporal variations in factors influencing device performance.^30^ Although individual devices are most often proprietary intellectual property, because of the time and expenses borne by diverse stakeholders, complex medical devices can be considered a shared commons, and achieving appropriate medical recalls is a key shared goal.

Solutions to mitigate device risks are not limited to removal from the market, with all the potential detriments of such action. Some devices can be rendered safe (or safer) by more nuanced changes such as improved labeling, warnings, instructions for use, software updates, component redesign, or alterations in power source. For example, after we reported a device risk associated with a Heimlich valve component of a chest tube kit, the manufacturer adopted verbatim our suggestion for a safety label.^1^ After we reported risk of a retained obturator component, the manufacturer also incorporated revised instructions (a banner emphasizing removal of the obturator) in an English-language training video (2 minute 33 second mark), although not in the Spanish, Italian, or German-language videos.^2^ ^,^ ^31^ Such interventions may be reasonable compromises given the costs (economic and other) to various stakeholders of outright removal of a device from the market.

A risk stratification ML algorithm could assist in identifying devices for such modifications, rather than complete product recall. The implementation of an ML approach and new data sources, concurrently with the techniques already employed by the FDA, might be a crucial aid in decreasing the intensive resources required for recall investigations, allowing the FDA to either investigate a wider breadth of devices or to focus more resources on devices that have a higher risk, thus potentially increasing the overall safety of the health field. Given the complexity of the medical device ecosystem and the early stage of our ML algorithm, we are not recommending that FDA recall devices be identified by an algorithm; rather, an ML algorithm could be used as part of a larger armamentarium to address risk.

Future work could characterize the predictive performance of an ML algorithm with even greater lead times, using additional data sources such as the FDA MAUDE database, and with prospective predictions for (as-yet) unrecalled devices. Even an algorithm that does not provide greater lead time than current FDA processes might increase efficiency and resource utilization of recall processes. Comparison of an ML algorithm’s warning performance with existing FDA processes, accuracy, and resource utilization would be meaningful next steps. The addition of an ML algorithm to existing FDA processes (rather than replacement of extant processes) is another potentially useful application.

## LIMITATIONS

Despite promising results, our study revealed several limitations and challenges of using ML in healthcare. The algorithm relies on Google Trends and PubMed footprints to increase its accuracy, meaning it is severely limited to devices with a significant online presence. Future iterations of the algorithm might include other free online information, such as the information in the MAUDE database, which was not incorporated into the current ML system. The lack of interpretability of ML algorithms makes it difficult to understand the underlying mechanisms behind their predictions, posing challenges in assessing their reliability and validity. An example is that while the algorithm can predict the FDA recalls, it is unclear whether it can determine if the device is dangerous for patients or will be recalled for other reasons. Another area that further challenges the reliability and validity is the content of PubMed or Google Trends searches. Authors could have only briefly mentioned a device; however, a search still yields it. Additionally, currently there is not a way to distinguish whether the mentions regarding a device are positive or negative, an area for future ML development.

The relatively small dataset of 500 devices may limit the generalizability of the algorithm and lead to a wide CI regarding both sensitivity and specificity. Future studies should consider using larger and more diverse datasets to train and evaluate the ML algorithm. Additionally, the algorithm applies FDA determinations as the reference standard. It remains to be proven that this source is the best comparator for the output provided by the algorithm since the FDA might be recalling devices unnecessarily or failing to recall dangerous devices. Although it did not occur commonly in our sample, an algorithm would be judged as failing if it did not match the FDA, even if in truth it were superior to the FDA process in recognizing dangerous devices. Our study only focused on predicting medical device recalls and did not evaluate the clinical effectiveness or safety of the devices themselves. We did not compare our algorithm performance to current FDA processes. Additionally, given how closely the ML algorithm performance matches that of the FDA, the FDA may already be using ML algorithms or similar processes.

## CONCLUSION

A machine learning algorithm using PubMed and Google Trends data predicted medical device recalls by the FDA with high sensitivity, specificity, and accuracy with lead times as great as 12 months. An ML algorithm might improve patient safety by enhancing the early detection and prevention of medical device recalls. Further research is needed to improve sensitivity, extend the forecasting window, and promote the development of ML algorithms in other healthcare segments, such as food and drug safety.

## Supplementary Information





