# Supplementary material for: A Machine Learning Algorithm to Predict Medical Device Recall by the Food and Drug Administration
Source: West J Emerg Med. 2024 Nov 21;26(1):161–70. doi: 10.5811/westjem.21238 (PMC11908527; doi:10.5811/westjem.21238)
Supplement: Supplementary file 2 [file wjem-26-161-s002.pdf]

device name
Accunet Embolic
AcrySof IQ PanOptix
AFFINITY NT OXYGENATION
AFFINITY PIXIE OXYGENATION
Allergan Natrelle Biocell
AQUAMANTYS Sealer
Armada 18 PTA
Armada 35
Atellica IM HBc Total 2
Auto CAT 2
STANLEY Automatic Doors
Bellafill Filler
Biocryl Rapide
CADD Infusion system
Carto Thermocool
CartoPrime
CereLink
Cerenovus Nimbus
Crescent RA Jugular Dual Lumen Catheter
Crescent Jugular Dual Lumen Catheter
Dermabond Advanced
Echelon 3000
Emboshield NAV6
ENDOPATH XCEL Trocars
Endurity Pacemaker
Fast-Cath Introducer
FLEXIPATH Trocars
FoundationOne
FoundationOne CDx
Fujifilm FDR
Hamilton C2
Hawkone
HeartSync Multifunction Disposable Single-Use AED
Heli-FX EndoAnchor
HomeStream Remote Management
icast stent
ICU Plum 360
Inogen Mini ICD
JJ Air Pen Driver
JUVÉDERM VOLUMA XC
JUVEDERM VOLUX
Ki-67 IHC MIB-1 pharmDx
Lava Liquid Embolic
LCP Distal Femur
LIAISON QuantiFERON-TB Gold Plus
LIAISON XL MUREX
LIAISON XL MUREX Control HCV Ab
LigaSure Maryland

Lucas CPR
Medtronic Cobalt
Medtronic Compia
medtronic synchromed ii
Medtronic Viva
Mentor Spectrum
Micrusframe S microcoil
Minimed Paradigm
MixeVac III
NeuRx Diaphragm
NovoTTF
ORBERA INTRAGASTRIC BALLOON SYSTEM
Oxylog 3000
Penumbra Jet 7
PerClot Polysaccharide Hemostatic
GE PET scan
Philips CPAP
Plasma Delipidation System
Precision7 for Astigmatism
PrisMax System
Prowler microcatheter
Puritan Bennett 980
RADIESSE (+)
Rapidsorb fixation
RelayPro Thoracic Stent
RHA Redensity
Ryoei CT
Sculptra
StarClose SE Vascular
Stellar 100
Stellarex Ballon
Stretcher
Stryker Cystoscope hardware
Stryker Laser cystoscope system
Superion InterSpinous Spacer
Thermedx FluidSmart Fluid management
Tight-N Tendon
TOPS System
TotalVisc Ophthalmic
TPLO System
TRILURON
ValleyLab FX8
Ventana Folr 1
VENTANA PD-L1
ventilators
Vercise DBS System
VertePort X4 Manifold cement
Vysis ALK
GE X-ray machine

Aveir DR Pacemaker
DiamondTemp ablation
Quadra Allure MP
980 ventilator
Abbott Amplatzer
Abiomed Impella
Absolute Pro Vascular Stent
AccelStim Bone Growth Stimulator
Access badges
Accolade MRI
Acculink Carotid Stent
AccuVein AV500
Acrion Saw Blade
AcrySof IQ PanOptix Toric
ACT PLUS AUTOMATED COAGULATION TIMER
ActaStim-S Spine
ACTIS Hip
Acuson AcuNav
Adonis AE 100
ADVIA Centaur HBc Total 2
ADVIA Centaur HBc Total 2 Quality Control
Advisor HD Grid Mapping
AFFINITY AF100 ARTERIAL
AFFINITY FUSION OXYGENATION
AFFINITY VENOUS AIR REMOVAL DEVICE
Agilent Resolution ctDx FIRST
Agili-C
Aglis HisPro
Aglis NxT
AITA Ethicon
Alaris pump
Alinity m CMV Assay
Allengers MARS 15
Amplatzer Amulet
Amplatzer Duct
Amplatzer Piccolo
Amplatzer Septal
Amplatzer Talisman
Amplatzer VSD
AMS X ray
ANSPACH EG1
ANSPACH EMAX 2 PLUS
ANSPACH XMAX
AOX Polyethylene
Aptima CMV Quant Assay
Arctic Front Advance
Arctic Front Advance Pro
Armada 14 PTA
Arrow Dwell Catheter

Artisyn Y-shaped mesh
Assert-IQ Monitor
Assurity Pacemaker
Atellica IM HBc Total 2 Quality Control
ATTUNE AFFIXIUM Knee
ATTUNE Cementless knee
AUTOLOG IQ AUTOTRANSFUSION
AVAflex balloon
Aveir Delivery Catheter
Aveir Leadless Pacing System
Aveir Link Module
Averir VR Leadless
Avive AED
Axonics Sacral System
Bandages
BAROSTIM NEO
Baxter Sigma Pump
Baxter Sigma Pump
BD Hypak Syringe
BearCare Walnut Wearable Smart Thermometers
Bellavista ventilator
Bioconsole 560
BioFreedom Stent
Biopatch protective disk
BioPrep Bone Preparation
Blake Drains
Blood work
Bodyguard infusion pump
Boston Scientific Semi-rigid autoclavable ureteroscope
Bovie MI 1000 Dual Surgery Light
Bovie MI-750 LED Procedure Light
CALCIVIS Imaging System
Caprosyn Monofilament Suture
Cerebase Sheath
Cerene Cryotherapy
Chocolate Touch Paclitaxel
Clamps
Coated Vicryl Suture
Cobalt XT
Cobas HBV
ConchaSmart
ConMed PadPro Electrodes
Cool-Tip RF Ablation
Corail system
Cordis Precise
CraniSeal Dural Sealant
Crocs
Crome ICD
CrossCath

CT scan
DASH Insulin
Decanav catheter
Defibrilators
Dermabond Prineo
DETOUR System
Disposable gloves
Draeger Carina
Draeger Seattle
Dragonfly OpStar Imaging
DreamStation
Durata Defibrilation Lead
DYNAGEN Mini ICD
Echelon Circular
ECHELON CONTOUR
Echelon Endopath
ECHELON FLEX ENDOPATH
Echelon Flex stapler
Echelon Gripping Surface Relaod
eCoin Peripheral Neurostimulator
EL SERIES BLOOD COLLECTION
electric scalpel
Electronic Scale
Ellipse Defibrillator
EMBLEM MRI S-ICD
EMBLEM S-ICD
Emboguard Catheter
EMBOTRAP II Device
Embotrap III revascularization
EMBOVAC Catheter
Emprint SX Ablation
Endoloop Ligature
ENDOPATH BASX Trocars
ENDOPATH DEXTRUS Minimally Invasive System
Endopath insufflation needles
Endurant II Stent
Engage Introducer
Engage TR introducer
ENROUTE Stent
ENSEAL X1 Straight Jaw Tissue Sealer
EnSite Precision
Enterprise 2 Vascular
Entrant HF CRT-D
Entrant ICD
Epic Max Stent
Epic Systems
Essentio MRI
Eterna SCS
Eversense E3 CGM

Eversense Glucose Monitoring
EVO Visian Implantable
EVO+ Visian Implantable
Evoke SCS
Exablate Neuro
EXALT Model B
FlexAbility Ablation
FreeStyle Libre
FreeStyle Libre 14 day
FreeStyle Libre 2 Flash Glucose
FRN-ADVANCED Femoral Nailing System
GALAXY G3
Galaxy G3 Mini
Galaxy G3 XSFT
GE carescape
GE carestation
GE Nuclear medicine 600
GE Nuclear medicine 800
GE TruSignal
Girraffe Incubator
GORE TAG Thoracic Branch
GRIPTION TF Acetabular
Guardant360
Hamilton C1
Hamilton C3
Hamilton C6
Hamilton T1
HEALIX ADVANCE Anchor
HEARTSPAN Needles
HeartStart FRx Defibrillator
HeartWare HVAD
Heliostar Catheter
Hemostats
Hintermann Series H3
HMS PLUS HEMOSTASIS
Hoka Shoes
Hospital Stretcher
Hudson RCI
IC-8 Apthera IOL
iCAST Stent
ICU Plum A+
ICU Plum A+3
Impella 5.5
Impella RP Flex
Infinity DBS
Infinity IPG
Ingenio Pacemaker
Inspire Upper Airway Stimulation
Insulet Omnipod

Interpulse Lavage System
INTERSEPT CARDIOTOMY RESERVOIRS
IonicRF Generator
Ivenix Pump
Jelco Hypodermic Needle
JJ Headless Scews
JVAC Reservoirs
Kendall Multifunction Defibrillation
KingFisher Presto
LCP T-Plate
Leica DMi8 Inverted LED
Leica VT1000 S
LIAISON Control QuantiFERON-TB Gold Plus
LIAISON XL MUREX HCV Ab
LifeSparc
LigaSure Blunt Tip
Lights
LiquiFix FIX8 Laparo
LungFit PH
M6-C Disc
Maestro Health
Magtrace
Mahurkar
Mallinckrodt One-Way Valve
MANTA Vascular
Medfusion 3500
Medfusion 3500
Medfusion 4000
Medtronic Amplia
Medtronic Brava
Medtronic Claria
Medtronic CoreValve
Medtronic Evera
Medtronic NIM
Medtronic Valleylab Electrosurgical Pencil
Medtronic Visia
Megadyne MEGA 2000
Mentor CPG
Mentor Saline Implant
Mentor Siltex
MENTOR CPX4
Mighty Bliss Electric Heating Pads
Mini Trek Catheter
Minimally Invasive Deformity Correction
Minimed 508
MiniMed 780G
Minitouch 3.8 Era
MiSight 1 Day Soft
MitraClip NT Clip

MitraClip NTR/XTR
Momentum EL
GE MRI
Multiparameter monitor
MYOTHERM XP CARDIOPLEGIA DELIVERY
Myriad myChoice
Nautilus Smart ECMO Module
Nautilus ECMO Oxygenator
Neuroform Atlas Stent
NeuroSphere Abbott
NeVa VS
Nit-Occlud PDA
Nitrile Gloves
NORMOFLO
NOxBOXi Nitric Oxide
Nucleus 24 Cochlear Implant System
Numeta G13E
Omnilink Elite Stent
Oncomine Dx
Oncomine Dx Target Test
Onyx Stent
Optilume BPH
Optilume Urethral Balloon
OPTIMIZER Smart System
OPTIS System
Organ Care System (OCS) Liver
Organ Care System Heart
OrganOx metra System
Osseonachored Prostheses
Otoscope
Oxygen catheter
Paramed Blood Pressure Monitor
PASCAL Precision Transcatheter
Patient Specific Talus Spacer
PD-L1 IHC 28-8
MEDLINE SURGICAL MARKER
Perciva ICD
Perclose ProGlide
Philips Bipap
Philips Forte Gamma
Philips respironics trilogy
PLASMABLADE Device
Portico Transcatheter
Portico with FlexNav TAVI System
Precision7
Precision7 Multifocal
Precision7 Multifocal Toric
PressureWire Receiver
Proclaim IPG

Proclaim Spinal Cord
Proclaim XR Spinal Cord
Prodigy Spinal Cord
Prometra Pump
Promus Elite
Promus Premier
Prospera Spinal Cord Stimulation
Prowler EX microcatheter
Proximate cutter
PROXIMATE Staplers
Pulse oximeter
Pulserider Aneurysm
QDOT Micro Catheter
Quantien Integrated Measurement
Quarter Quadripolar LV
Radiesse Injectable Implant
RealTime HCV Genotype II Assay
Rebel Platinum Stent
REFLECT Scoliosis
Regent Mechanical Valve
Reliant Stent
Resilience Percutaneous Lead
RESMED Stellar 150
Resolute Onyx Stent
Restylane Eyelight
Restylane Lyft
Revanesse Lips+
Revolution Cement Mixer
ROSA One
Rotarex Rotational Atherectomy
Saline solution
Sangia Total PSA Test
SBL-3 Lens
scalpel
Scoreflex Cathether
Sentimag
Sentrant Introducer Sheath
Senza Spinal Cord Stimulation
Shockwave Intravascular Lithotripsy
Siemens CT
SKINVIVE by JUVÉDERM
SmartPump Tourniquet System
SoftVue Automated
SoloAssist II Robotic Scope Holder
Sonalleve MR-HIFU
Sonicision 7 Curved
Spanner Stent
Spatz3 Adjustable Balloon System
S-ROM NOILES Rotating

StableVisc Device
STAR S4 IR Excimer Laser
stethoscope
Stryker Flexible instruments
Stryker Hospital Bed
Stryker Procedural Oxygen Mask
Supera Stent
SurVeil Ballon
Synergy Megatron Stent
SYNERGY Platinum Stent
TactiCath Quartz Catheter
Tactiflex Ablation Catheter
TAD Tapered Guide Wire
Tendril Pacing Lead
The Freezor Max
The Tether System
ThermaCor 1200
ThermoBrite System
Thermometer
TherOx DownStream
Thoraflex Hybrid
Tibial Tuberosity Advancement System
TigerWire guide
Toshiba CT
TourGuide Steerable Sheath
Trek catheter
Trilogy EV300
Trilogy EVO
Trilogy EVO O2
Trilogy EVO Universal
Trufill n-BCA
TruSignal
Tula System
tuttnauer autoclave
Tweezers
UroVysion
v60 ventilator
VALITUDE X4
ValleyLab FT10
Valleylab Laparoscopic Smoke Evacuation
ValleyLab LS10
Variable angle LCP
VEGA Endocardial
Velosorb Braided Suture
Venom cannula and electrode system
VENTANA ALK CDx Assay
VENTANA MMR
Vercise Gevia
Vercise PC

VICI VENOUS STENT
VIP 2000
VNS therapy
Volara system
Watchcare incontinence management system
WATCHMAN FLX
WATCHMAN FLX Pro
Wheelchair
xT CDx
Zilver Stent
Zoom 71
